# Utilization of Suberinic Acids Containing Residue as an Adhesive for Particle Boards

**DOI:** 10.3390/polym14112304

**Published:** 2022-06-06

**Authors:** Raimonds Makars, Janis Rizikovs, Daniela Godina, Aigars Paze, Remo Merijs-Meri

**Affiliations:** 1 Latvian State Institute of Wood Chemistry, Dzerbenes iela 27, LV-1006 Riga, Latvia; janis.rizikovs@kki.lv (J.R.); daniela.godina@kki.lv (D.G.); aigars.paze@kki.lv (A.P.); 2 PolyLabs SIA, Mukusalas iela 46, LV-1004 Riga, Latvia; 3 Institute of Polymer Materials, Riga Technical University, Paula Valdena iela 3/7, LV-1048 Riga, Latvia; remo.merijs-meri@rtu.lv

**Keywords:** birch outer bark, suberinic acids, particle boards

## Abstract

The birch (*Betula* spp.) outer bark is a valuable product rich in betulin. After removal of betulin extractives, suberin containing tissues are left. Suberin is a biopolyester built from α,ω-bifunctional fatty acids (suberinic acids), which after depolymerization together with lignocarbohydrate complex is a potential adhesive as a side-stream product (residue) from obtaining suberinic acids for polyol synthesis. In this work, we studied the utilization possibilities in particleboards of the said residue obtained by depolymerization in four different solvents (methanol, ethanol, isopropanol and 1-butanol). The adhesives were characterised by chemical (acid number, solubility in tetrahydrofuran, epoxy and ash content) and instrumental analytical methods (SEC-RID, DSC, TGA and FTIR). Based on the results of mechanical characteristics, ethanol was chosen as the most suitable depolymerization medium. The optimal hot-pressing parameters for particleboards were determined using the design of experiments approach: adhesive content 20 wt%; hot-pressing temperature 248 °C, and hot-pressing time 6.55 min.

## 1. Introduction

Birch (*Betula* spp.) trees are very common in the Northern hemisphere. Veneer production is considered the most efficient way of processing birch wood, and birch bark is accumulated as the by-product in this process and mostly used for the production of thermal energy by incineration. Birch bark is composed of two layers: the outer bark and the inner bark [1]. The outer bark proportion of the total birch biomass is roughly 3 to 5 wt% [2]. Although the amount of bark is relatively small, the large production volumes of veneer result in a noticeable accumulation of this material.

The birch outer bark (BOB) contains a high proportion of lupane-type triterpene extractives (betulin, lupeol, betulinic acid) which can be isolated by various methods [3,4,5]. After the removal of extractives from BOB, mainly suberin-containing external protective tissues are left [6].

Suberin is a biopolymer of plant origin occurring in specific parts of tissue, where protection from the surrounding environment is required. Chemically, suberin comprises two chemically distinct parts: (a) an aliphatic region built from α,ω-bifunctional fatty acids (suberinic acids (SA)) and glycerol through ester linkages; (b) polyaromatic lignin-like structures [7]. These regions are cross-linked and various depolymerization methods are proposed to obtain SA monomers and oligomers. The most common method is alkaline hydrolysis in water [8,9] and other solvents, such as isopropanol [2,6]. Our previous research showed that SA obtained by hydrolytical depolymerization can be further used as a bio-based adhesive in the preparation of environmentally friendly wood composites, such as plywood and particle boards. The particle boards made from SA-based adhesive in hot-pressing process exhibited satisfactory water-resistant properties as determined by low thickness swelling values. In addition, the high internal bonding values showed that the SA-based adhesive has as good compatibility with wood particles as a filler [9,10]. The satisfactory water resistance is explained by the lipophilic properties of suberin [11].

In addition, in our previous work [12] we have demonstrated that SA can be obtained from extractive-free BOB by alkaline depolymerization in ethanol-water solution for further utilization in polyol synthesis. During this process, residue containing SA and lignocellulosic components is accumulated. One way to utilize this residue after suberin depolymerization is as solid fuel or fuel filler [13]. However, these products have low added-value, therefore the aim of this study was to investigate the utilization of the residue obtained after depolymerization in four different solvents (methanol, ethanol, isopropanol and 1-butanol) in particle board preparation. For the most promising adhesive, the design of experiments (DOE) approach was used to determine the optimal hot-pressing parameters.

## 2. Materials and Methods

### 2.1. Raw Material—Birch Outer Bark

Isolated and fractionated BOB was kindly supplied by AS Latvijas Finieris (Riga, Latvia). BOB samples were dried at room temperature (moisture content 4–5 wt%) and milled in an SM 100 cutting mill (Retsch GmbH, Haan, Germany) to pass through a sieve with holes measuring 4 mm in diameter. Milled BOB was fractionated by sieving using an AS 200 Basic vibratory sieve shaker (Retsch GmbH, Haan, Germany). Fraction of >4 mm was collected and repeatedly milled in the same cutting mill to pass through a sieve with 2 mm aperture. BOB was further extracted with ethanol twice, as described by Godiņa et al. [14]. Extracted BOB was dried at room temperature and further used as a feedstock for depolymerization.

### 2.2. Other Materials and Chemicals

Ethanol (96,3% *v*/*v*) was supplied by SIA Kalsnavas elevators (Jaunkalsnava, Latvia). Methanol (≥99.8%), hydrochloric acid (HCl) (≥37%), and tetrahydrofuran (THF) (anhydrous, ≥99.9%) were obtained from Sigma-Aldrich (Steinheim, Germany). Isopropanol (Reag. Ph Eur, ≥99.8%) was received from Merck (Darmstadt, Germany). 1-Butanol (99%) was purchased from Acros Organics (Geel, Belgium). Potassium hydroxide (KOH) (Reag. Ph Eur, 85.0–100.5%) was provided by VWR International (Leuven, Belgium). Nitric acid (HNO_3_) (≥65%) and acetone (≥99%) were obtained from Honeywell (Seelze, Germany).

### 2.3. Obtaining SA-Based Adhesive

BOB depolymerization was carried out in KOH solution for 60 min at 66 to 80 °C (depending on the solvent). Four different solvents were used: methanol (MeOH), ethanol (EtOH), isopropanol (i-PrOH) and 1-butanol (BuOH). The depolymerization conditions are given in Table 1.

After depolymerization, the solution was cooled down and filtered. The filtrate can be further used for preparing polyols as described by Rizikovs et al. [12]. The precipitate (residue) was further used to prepare the adhesive by suspending it in water to the ratio of 100 g per litre. The suspension was acidified with HNO_3_ to pH 2.0, then it was filtered and rinsed with deionized water. As a result, four different SA-based adhesive samples were obtained.

### 2.4. Characterization of SA-Based Adhesive

For SA characterization, adhesive samples were dried at room temperature and milled with a CryoMill cryogenic mill (Retsch GmbH, Haan, Germany) under LN_2_ purge at −196 °C.

#### 2.4.1. Acid Number

To about 0.2 g of the sample, 5 mL of DMSO was added and stirred for 1 h. Afterwards, 20 mL of i-PrOH and 5 mL of water were added, and the solution was titrated with 0.1 M KOH solution. Two replicate experiments were performed for each sample.

#### 2.4.2. Epoxy Groups

To about 0.2 g of the sample, 10 mL of 0.2 M HCl in acetone was added. The solution was stirred for 1 h and titrated with a known concentration of 0.1 M KOH solution. Two replicate experiments were performed for each sample.

#### 2.4.3. Soluble Substance in THF

To about 0.1 g sample 5 mL of THF was added. The mixture was heated at 50 °C for 0.5 h, cooled and stirred for another hour. The soluble substance in THF was determined by filtration through a filter crucible (porosity 2). Two replicate experiments were performed for each sample. The filtrate was further used for size exclusion chromatography analysis.

#### 2.4.4. Ash Content

The ash content was determined at 715 ± 10 °C according to ISO standard 1171:2010 [15].

#### 2.4.5. Size-Exclusion Chromatography

GPC analysis of sample (100 μL) was performed using an Agilent Infinity 1260 HPLC system with degasser, auto sampler, RI detector and MALS (miniDAWN) detector. Two GPC analytical columns connected in line were used for the analysis: PLgel Mixed-E (3 uL, 300 × 7.5 mm). The flow rate was 1 mL/min and the RI detector temperature was 35 °C.

The polystyrene calibration graph was obtained by preparing polystyrene standard solutions in THF at a mass concentration of 2 mg/mL and analysing them with GPC instrument. Molecular weights of polystyrene standard substances were 500, 850, 1000, 2500, 5000, 9000, 17,500, 20,000, and 30,000 Da.

#### 2.4.6. Differential Scanning Calorimetry (DSC)

DSC822 differential scanning calorimeter (Mettler-Toledo, Greifensee, Switzerland) was used to analyse the thermal behaviour of adhesive samples. The analysis was performed in pierced aluminium pans under N_2_ purging at a rate of 10 °C min^−1^ in two stages: (1) heating from 20 °C to 260 °C; (2) cooling from 260 °C to 20 °C.

#### 2.4.7. Thermogravimetric Analysis (TGA)

TGA analysis was performed using a TA Instruments Discovery TGA 5500 thermogravimetric analyzer. Mass loss was determined in Pt sample pans under an N_2_ purge at 50 mL min^−1^ by isothermally treating the sample at 30 °C, followed by heating to 700 °C at a rate of 10 °C min^−1^.

#### 2.4.8. Fourier Transform Infrared Spectroscopy (FTIR) Analysis

FTIR spectrometry data was collected with a Nicolet iS50 spectrometer (Thermo Fisher Scientific, Waltham, MA, USA) at a resolution of 4 cm^−1^, 32 scans. The FTIR data were collected using the attenuated total reflectance (ATR) technique with a diamond crystal prism.

### 2.5. Particle Board Preparation

The SA-based adhesive with moisture content ∼80 wt% was mixed with fractionated (0.4–2.0 mm) birch wood particles generated from veneer shorts. Afterwards, the mixture was oven-dried at 100 °C to moisture content <1 wt% and hot-pressed with thickness bars by a LAP 40 single-stage press (Gottfried Joos Maschinenfabrik GmbH & Co. KG, Pfalzgrafenweiler, Germany). The dimensions and density of the boards were designed to be 180 × 150 × 7 mm and 0.8 g cm^−3^, respectively.

#### 2.5.1. Comparison of Particle Board Properties Depending on SA-Based Adhesive Sample

To choose the most suitable adhesive, MeOH, EtOH, i-PrOH, and BuOH adhesive-based particle boards were made, as described above. Based on previous experience [9] particle board hot-pressing temperature (T) was set to 225 °C and the pressing time (t) was 5 min. Pressure (p) was varied in two cycles: 3.5 MPa in the first cycle (t = 2 min) followed by pressure release to 0.1 Mpa per 30 s; during the second cycle, the pressure was increased to 1.7 Mpa (t = 1 min 50 s) followed by another pressure release to 0.7 Mpa for 40 s. The adhesive content (c) in the particle boards varied from 20 to 40 wt% (dry basis).

#### 2.5.2. Experimental Design to Determine Optimal Hot-Pressing Parameters

After choosing the most suitable adhesive, the design of experiments (DOE) approach was used to determine the optimal hot-pressing parameters. Three variable factors were defined: c, T, and t. At first, full factorial design methodology (2^3^) using Design Expert 13 software (Stat-Ease Inc., Minneapolis, MN, USA) was used, which consisted of 8 runs. The variable factor levels are given in Table 2. Depending on t, the hot-pressing was performed in 2 or 3 cycles (at t = 2 and t = 8 min, respectively). The duration of the hot-pressing cycles is given in Table 3.

The effects of the hot-pressing parameters on response values (modulus of elasticity (MOE), bending strength (MOR), and thickness swelling after a 24 h immersion test (TS 24 h), density) were evaluated using the software. To improve the resolution, the full factorial experimental plan was augmented by 8 additional runs using D-optimality criteria.

### 2.6. Evaluation of the Particle Board properties

The obtained PBs were conditioned (RH = 65 ± 5%, T = 20 ± 2 °C) and characterized according to relevant standards by density [16], MOE and MOR in the 3-point bending test [17], and TS 24 h [18]. The particle boards obtained using the optimal hot-pressing parameters were characterized by internal bonding (IB), determining the tensile strength perpendicular to the plane of the board [19]. Mechanical tests (MOE, MOR, IB) were performed on a Z010 (Zwick Roell AG, Ulm, Germany) universal machine for testing the resistance of materials. The obtained particle boards were compared with the EN 312 standard requirements [20].

## 3. Results and Discussion

### 3.1. Characterization of SA-Based Adhesive Samples

#### 3.1.1. Acid Number, Epoxy Group Content, Soluble Substance in THF, Ash Content

Chemical analysis (acid number, epoxy group content, soluble substance in THF, ash content) was carried out to determine the properties of adhesive samples. The results are summarized in Table 4. Acid number values show that the highest acid functionality was for i-PrOH samples (122 mg KOH g^−1^), which could be a desirable characteristic for an adhesive, because acid groups are involved in adhesive cross-linking reactions. However, the i-PrOH adhesive had the lowest epoxy group content (0.11 mmol g^−1^). A high epoxy group content is a desirable property since epoxy groups play a role in the formation of cross-linked networks [21]. From that point of view, the epoxy group value for the EtOH adhesive was almost 6 times higher—0.61 mmol g^−1^. i-PrOH and BuOH adhesives showed higher values for the soluble substance in THF (58.1 and 57.5 wt%, respectively), compared to the MeOH sample (44.0 wt%), which suggests that a higher proportion of suberin-based monomers and oligomers were present in those samples. On the contrary, the MeOH sample had a relatively high ash content (12.9 wt%). This could be because of the higher SA particle aggregation in the acidification process. As a result, unacidified SA salts remained in the agglomerates, so the adhesive potentially could be less effective. The lowest ash content values were for SA obtained in the BuOH and EtOH (6.6 and 6.7 wt%, respectively).

Because of the high amount of acid number, epoxy groups, soluble substance in THF, and the low ash content adhesive obtained in ethanol showed the most promising properties for obtaining wood-based panels.

#### 3.1.2. Size-Exclusion Chromatography

**Figure 1:**
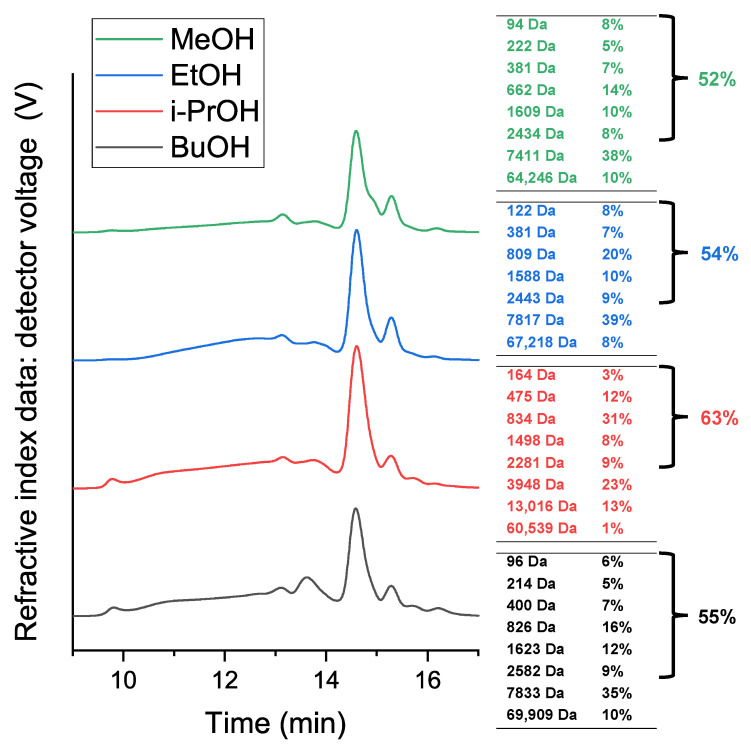
SA-based adhesive sample SEC-RID chromatograms.

The adhesive molecular weight is also an important characteristic of the adhesive. During SEC-RID analysis of the soluble substance in THF, seven to eight (depending on the sample) different molecular weight fractions were separated, ranging from 94 to 69,909 Da. Since adhesives have limited solubility in THF, it is possible that larger molecular weight fractions (including lignocellulose) were left out. The chromatograms with the relevant molecular weight fractions are given in Figure 1. For a more convenient result comparison, lower (oligomeric + monomeric) molecular weight fraction (<3000 Da) percentages were estimated.

Overall, the SEC-RID analysis shows similarities in the separation patterns. The analysis showed that the highest low molecular weight portion was for the i-PrOH sample, which corresponds to the higher acid number for the sample (Table 4). In addition, the solubility in THF was the highest and the M_n_ and M_w_ values were the lowest for this sample (Table 5), both indicating that this adhesive contained a higher proportion of low molecular weight fraction. Consequently, the polydispersity was higher for MeOH, EtOH and BuOH adhesives. The high polydispersity values show that a variety of monomers and oligomers were represented in all the samples. Suberin’s aliphatic structure mostly comprises C_16_ to C_24_ α,ω-diacid and ω-hydroxyacid monomers [7] with molecular weights ranging from around 270 to 400 Da. Thus, the most pronounced peak at t_R_ ≈ 14.5 min for molecular weights ranging from 662 to 834 Da is most likely attributed to SA dimers in the adhesives.

#### 3.1.3. DSC Analysis

**Figure 2:**
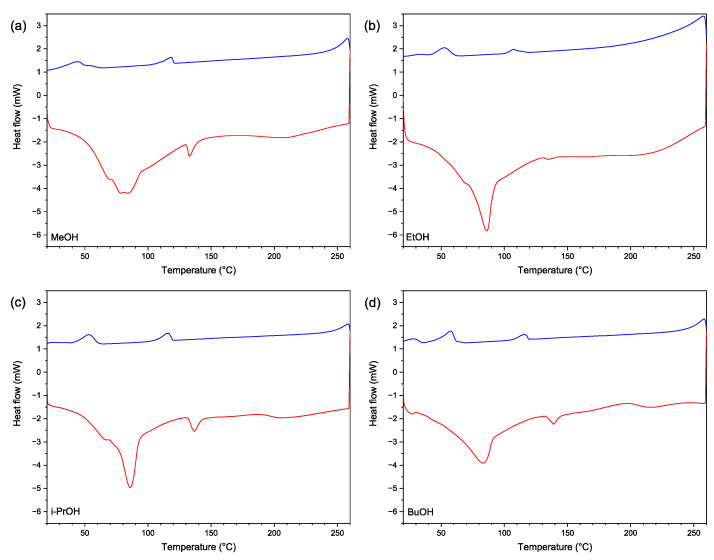
DSC thermograms for (**a**) MeOH, (**b**) EtOH, (**c**) i-PrOH, and (**d**) BuOH adhesive samples (the red curve represents heating from 20 °C to 260 °C and the blue curve represents cooling from 260 °C to 20 °C).

**Figure 3:**
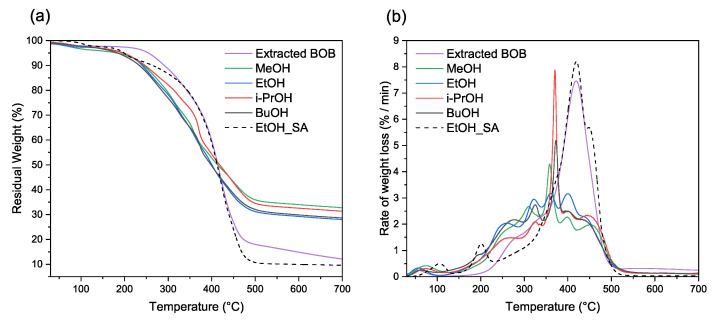
TGA (**a**) and DTGA (**b**) curves for adhesive and feedstock samples.

DSC thermograms in Figure 2 show that all four adhesives have two common crystalline phase melting zones (see red curve) at 84–87 °C and 133–139 °C. Heating curves for MeOH, EtOH and i-PrOH samples also had an additional shoulder at 65–70 °C, and for the MeOH adhesive, an additional peak at 78 °C was observed. Both EtOH and i-PrOH samples exhibited more pronounced crystalline phase melting areas, showing that more crystalline SA were present on these samples. The acid number values were also higher for these samples (Table 4) and this may suggest that acidic groups form more crystalline structures, most likely due to intermolecular hydrogen bonding. The cooling curves (in blue) showed crystallisation zones of the corresponding melting peaks. The areas of the corresponding crystallisation peaks at around 110 °C and 50 °C were smaller than the melting peaks, which could indicate to the thermal destruction (see TGA curves in Figure 3) and to intramolecular reactions in the heating process, and, thus, resulting in cross-linked structures. This suggests that all adhesives have thermosetting properties, which is necessary for obtaining wood composites with desirable properties.

#### 3.1.4. TGA Analysis

As seen in the TGA DTGA curves in Figure 3, all the adhesive samples showed similar weight loss patterns. The first weight loss was observed below 100 °C due to the moisture content in the sample. Onset temperature values in TGA curves for the i-PrOH and EtOH adhesives were the lowest at 207.6 °C and 207.8 °C, respectively, whereas MeOH was more heat resistant with an onset temperature of 232.2 °C. The second decomposition in DTGA starts at around 200 °C, which could be attributed to the decomposition of suberin-like structures. This was confirmed by acquiring an additional TGA curve from pure SA obtained from the filtrate, as mentioned in Section 2.3. (sample EtOH_SA). In addition, there is a possibility that suberin mass loss over 200 °C temperature overlaps with lignin decomposition [22], resulting in a second peak at 255–277 °C. According to Şen et al. [23] the hemicellulose decomposition of *Betula pendula* bark samples occurs at 309 °C, which coincides with the third peak at 310–325 °C. A sharp peak at 358–373 °C is characteristic because of the degradation of cellulose [23] followed by a decomposition of suberin components at ∼400 °C and 443–450 °C, attributing to ω-hydroxy acids and α, ω-diacids respectively [22,23]. When comparing the DTGA curves of adhesive samples to feedstock (extracted BOB), there is a clear decrease in SA-related decomposition. This is due to the removal of some of the suberinic acids after depolymerization for further use in polyols.

Based on experimental findings from *Betula pendula* bark pyrolysis [23], suberin composition can be estimated using a multi-peak fitting technique of the DTGA signal. With the help of Origin Pro 2021b software deconvolution tool, we estimated the cellulose and suberinic acid content using Lorentzian multi-peak fitting. Aromatic suberin and lignin content were estimated from resulted char (at 700 °C) by subtracting ash content from the residue after TGA analysis. The results of chemical composition are summarized in Table 6. Cellulose content, as well as aromatic suberin plus lignin values, were close to all the samples. EtOH sample had the highest ω-hydroxy acid and total suberinic acid (ω-hydroxy + α, ω-diacids) content, which is important for the adhesive effectiveness.

#### 3.1.5. FTIR Analysis

**Figure 4:**
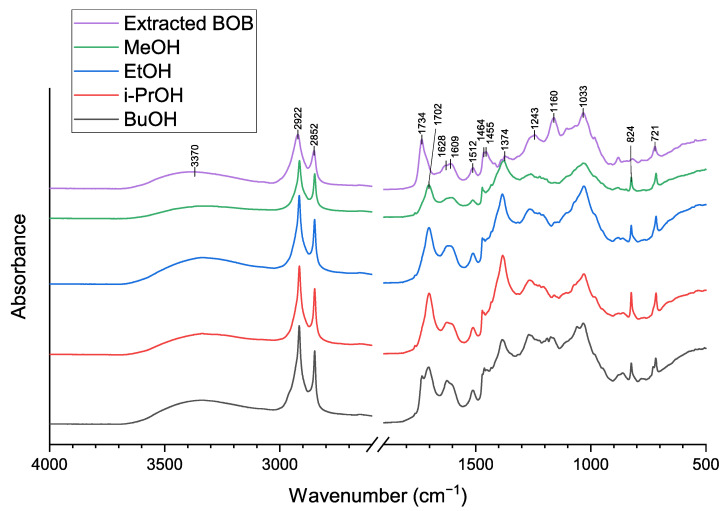
FT-IR spectra for adhesive samples and feedstock (extracted BOB).

Infrared spectra (Figure 4) for adhesive samples and feedstock (extracted BOB) showed a difference for C=O absorption bands. The band at 1734 cm^−1^ was attributed to esters, whereas the band at 1702 cm^−1^ corresponds to C=O vibration in carboxylic acids [24,25]; thus confirming that adhesive samples consisted mostly of depolymerized SA. Another band at 1160 cm^−1^ was present just for the feedstock sample. The C–O–C vibrations at this wavenumber were most likely attributed to ester linkages [26]. Only for BuOH sample, the peak at 1734 cm^−1^ was present, as well as a band at 1160 cm^−1^ was more pronounced than for other adhesives. This may suggest that 1-butanol was the least suitable solvent for BOB depolymerization. Asymmetric (2922 cm^−1^) and symmetric (2852 cm^−1^) vibrations, as well as bands at 1464, 1455, and 1374 cm^−1^ were another characteristic of suberin, corresponding to C–H bonds in alkyl chains [24,25,26,27]. The vibrations at 721 cm^−1^ are inherent to suberin resulting from aliphatic R_1_CH=CHR_2_ structures [24,27]. Absorbance at the 1243 cm^−1^ region can be attributed to epoxy groups in suberin structures and to cellulose and lignin [26,27]. It appears that this band was slightly less pronounced for the MeOH sample, although MeOH epoxy group content (Table 4) was higher when compared to i-PrOH and BuOH samples, confirming that there is an overlap in the epoxy, cellulose, and lignin signals.

Lignin and suberin aromatic components in the adhesive and feedstock samples were identified at 1628 and 1609 cm^−1^ resulting from C=C vibrations from the conjugated carbonyl groups of aromatic components. Absorbances at 1512 and 824 cm^−1^ are another characteristic band for lignin, corresponding to aromatic C=C stretching and ring vibrations [27]. The more pronounced band at 1628, 1609, and 824 cm^−1^ points to the fact that the lignin content has increased in the adhesive again, confirming the removal of some of the suberinic acids after depolymerization for further use in polyols. The peak at 1033 cm^−1^ is associated with C–O vibrations in polysaccharide components [24,27]. The stretching at around 3370 cm^−1^ can be attributed to O–H stretching in polysaccharides and due to the moisture in the sample.

### 3.2. Choosing the Most Suitable Adhesive for PB Hot-Pressing

To assess the properties of MeOH, EtOH, i-PrOH, and BuOH adhesive-based composites and compare their mechanical properties, particle board samples from the corresponding adhesives were made. The adhesive content was the only variable in these experiments (c = 20–40 wt%).

The data collected in Table 7 show that the density values for all the composites obtained were relatively similar and were just slightly higher than the designed density (0.830 g cm^−3^). The differences between the other properties of the particle boards were more noticeable. Higher TS 24 h value was achieved with higher adhesive content. TS 24 h requirements were met by all samples made from i-PrOH, BuOH and by two samples from EtOH adhesive (c = 30 wt% and 40 wt%). Overall, MeOH adhesive-based particle boards had the poorest mechanical properties. Regarding the MOE values, very good mechanical properties were achieved by composites based on EtOH and i-PrOH adhesives. BuOH adhesive-based composites showed satisfactory MOE values at c = 20 and 30 wt% when compared to standard P2 requirements. Overall, with an increase in adhesive content, the MOE value decreases for the particleboards, and, therefore, higher adhesive content corresponds to less stiff composites. MOR values are amongst the lowest for BuOH adhesive-based boards. EtOH and i-PrOH adhesive-based particle boards showed the highest values of MOR. However, none of them met the requirements of standards P2 and P3.

It seems that the higher acid number value (Table 4) corresponded to the overall better mechanical properties of the particle boards. The poor mechanical properties of MeOH adhesive-based boards could also be explained both by too little content of suberinic acids and their oligomers in the sample (low solubility in THF, Table 4) and because this adhesive had higher thermal stability, which requires a higher hot-pressing temperature. In addition, unwanted condensation reactions during the drying stage of adhesive and wood particle mixture may have resulted in low particle board mechanical properties.

In summary, the most perspective depolymerization mediums for obtaining particle board adhesive from BOB seem to be EtOH and i-PrOH. From a technological point of view, obtaining the EtOH adhesive was less complicated. In addition, the cooling of i-PrOH depolymerizate resulted in rapid precipitation. It seems that this precipitation was a reason that i-PrOH adhesive exhibited highest acid number (Table 4). However, the yield of SA that can be obtained from filtrate after depolymerization in an i-PrOH was about 18% lower when compared to EtOH-obtained SA, which is important for SA-based polyol synthesis [12]. Therefore, it is important to add that the SA-based adhesives that are studied in this paper are obtained as a side-stream product. Based on this evidence, the EtOH adhesive was considered the most suitable for further investigation of optimal hot-pressing parameters.

### 3.3. Experimental Design for Obtaining Particle Boards

As mentioned, the EtOH adhesive was chosen as the most suitable adhesive from the obtained data on the properties of the particle boards, for the full factorial experiment (FFE). The adhesive was further used to make particle board composite materials for investigation of the effects of the hot-pressing parameters on the properties of the particle boards through the FFE approach.

Depending on hot-pressing parameters, the properties (MOE, MOR, TS 24 h, density) of particle boards were studied. The results in Table 8 show that lower c and higher T and t values corresponded to better mechanical properties. It can be seen that temperature plays a very important role in the curing properties of SA-based adhesive, judging by MOE and MOR values. It also seems that the ability of the adhesive to penetrate the filler (wood particles) improves with an increase of the temperature, indicated by lower TS 24 h values. Overall, the statistical analysis showed that all hot-pressing variables had a significant effect on the particle board properties (*p* < 0.05).

To improve the resolution, the experimental plan was augmented with eight additional runs using D-optimality criteria. The mechanical properties of the obtained particle boards are summarised in Table 9. The boards with t = 2 min were hot-pressed according to the parameters given in Table 3. The rest of the boards were hot-pressed in two cycles: during the first cycle, the pressure was 3.5 MPa (t = 120 s) followed by a reduction in the pressure to 0.1 MPa for 30 s; in the second cycle, the pressure was 1.7 MPa (t = 50 to 180 s) with a subsequent pressure release to 0.7 MPa for 15 to 28 s.

Overall, the densities were slightly higher than the designed values. MOE values for most of the boards met the EN 312 P3 and P2 requirements, however, this was not the case with the MOR values. At this stage of the study, it seemed that the standard requirements P2 can only be met due to the comparatively low MOR value. In addition, for P2 requirements TS 24 h value was not relevant. However, most of the particle boards exhibited good water resistance (TS 24 h values below 15%) confirming the beneficial water-proofing properties of SA.

After summarizing the data, 4D mathematical models depending on the obtained results were developed and their visual representations are further discussed in the following subsubsections.

#### 3.3.1. MOE of the Particle Boards

**Figure 5:**
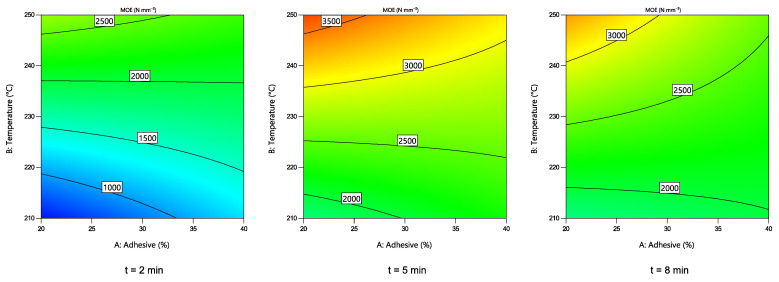
Changes in the MOE value of the boards depending on the hot-pressing parameters.

Changes in MOE of the obtained boards depending on the hot-pressing parameters are shown in Figure 5. A quadratic numerical model was chosen, as suggested by the software. To simplify the model, statistically insignificant values were excluded. According to ANOVA results, T, t, s … T, s … t, T … t and t^2^ variables had a statistically significant effect (*p* < 0.05) on the MOE value. It can be seen that higher MOE values can be achieved at higher hot-pressing temperatures. These results corroborate that the formation of more cross-linked structures, and, thus, higher stiffness can be achieved at higher temperatures. In addition, as the hot-pressing time increases from 2 to 5 min, the value of MOE increases, but it decreases as it approaches t = 8 min, suggesting that at prolonged hot-pressing periods the degradation of both wood particles and adhesive occur.

#### 3.3.2. MOR of the Particle Boards

**Figure 6:**
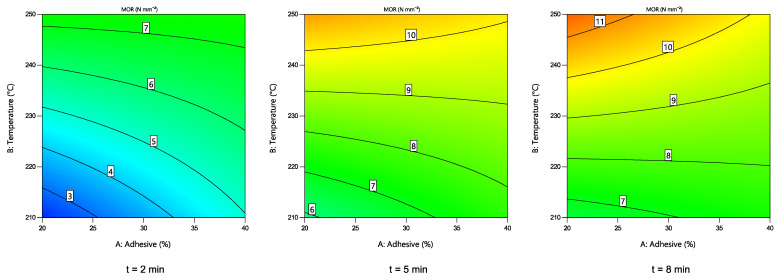
Changes in the MOR value of the boards depending on the hot-pressing parameters.

Changes in MOR of the obtained boards depending on the hot-pressing parameters are shown in Figure 6. A quadratic numerical model was chosen, as suggested by the software. To simplify the model, statistically insignificant values were excluded. According to ANOVA results, T, t, s … T, s … t and t^2^ variables had a statistically significant effect (*p* < 0.05) on MOR value. The graphic interpretation of the model shows that at higher hot-pressing times and higher temperatures, it is possible to reach higher MOR values at relatively low adhesive contents.

#### 3.3.3. TS 24 h of the Particle Boards

**Figure 7:**
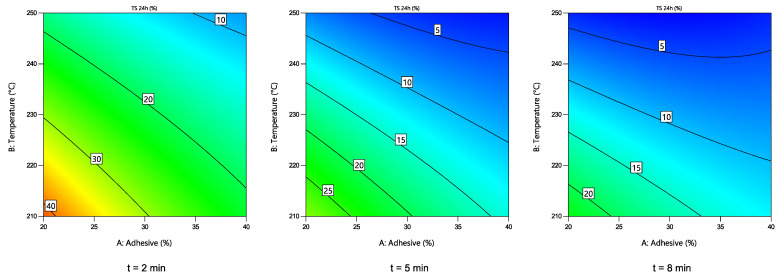
Changes in the TS 24 h value of the boards depending on the hot-pressing parameters.

Changes in TS 24 h of the obtained boards depending on the hot-pressing parameters are shown in Figure 7. A quadratic numerical model was chosen, as suggested by the software. To simplify the model, one statistically insignificant value (T^2^) was excluded. All the rest of the values showed statistically significant effects (*p* < 0.05) on the water-resistance of the boards, which is also shown in the graphic interpretation of the model. It can be seen that the hot-pressing time had a noticeable effect as the moisture resistance (lower TS 24 h values) was significantly improved at t = 5 min (compared to t = 2 min). After a further increase of duration to t = 8 min, the improvement of the 24 h value is less pronounced.

#### 3.3.4. Particle Board Density

**Figure 8:**
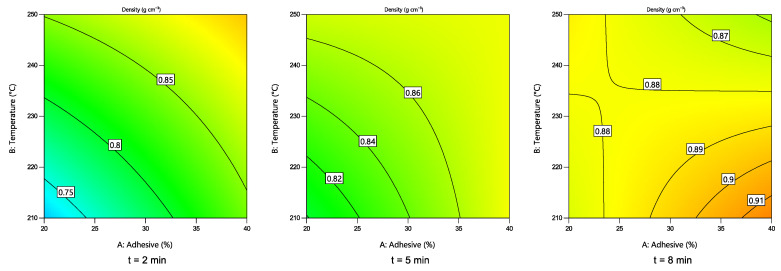
Changes in the density of the boards depending on the hot-pressing parameters.

Changes in the density of the obtained boards depending on the hot-pressing parameters are shown in Figure 8. The two-factor interaction numerical model was chosen, as suggested by the software. Statistical analysis showed that all hot-pressing parameters significantly (*p* < 0.05) affected the density of the boards. An interesting pattern can be seen for higher t values (8 min): at lower hot-pressing temperatures and higher c, the density is rather high. However, with an increase in T, the density further decreases. It could be explained by the rapid release of condensation and decomposition products at higher temperatures, resulting in lower density boards.

#### 3.3.5. Determination of Optimal Parameters for Particle Board Hot-Pressing

The constraints of the variables and response values given in Table 10 were used for the determination of optimal hot-pressing parameters.

After setting the goals, the software suggested the following optimal hot-pressing parameters: c = 20 wt%; T = 248 °C and t = 6.55 min. To confirm the results, particle boards were hot-pressed according to the suggested parameters in two cycles. During the first cycle, the pressure was 3.5 MPa (t = 150 s) followed by a reduction in the pressure to 0.1 MPa for 30 s; while in the second cycle, the pressure was 1.7 MPa (t = 180 s) with a subsequent pressure release to 0.7 MPa for 34 s. All four response values as well as additionally IB were determined for the resulting boards and the results are given in Table 11. It can be seen that all values were within the limits of the model’s 95% prediction interval and also met the EN 312 P2 requirements (Table 7).

## 4. Conclusions

The present work studied the possibilities of obtaining and utilising suberinic acid-containing residues after birch outer bark depolymerization in the alkanol environment. Four different adhesives obtained in four different solvents (methanol, ethanol, isopropanol, 1-butanol) were compared by chemical and thermal properties, as well as their performance in particle board bonding. The highest acid number (122.0 mg KOH g^−1^) was reached when the adhesive was obtained in isopropanol, but this adhesive was characterised with the lowest epoxy content (0.11 mmol g^−1^). For ethanol-based adhesive, the epoxy content (0.61 mmol g^−1^) and ω-hydroxy suberinic acid (17.5 wt%) content as determined with DTGA was the highest. TGA analysis also showed that all obtained adhesives showed heat resistance from 207 °C for adhesives obtained in ethanol and isopropanol to 232 °C (methanol adhesive). Based on particle board mechanical tests, ethanol was chosen as the most appropriate depolymerization medium for obtaining adhesive. The optimal hot-pressing parameters were determined using the design of experiments approach: adhesive content 20 wt%; hot-pressing temperature 248 °C and hot-pressing time 6.55 min. The obtained optimal particle boards met the EN 312 P2 requirements and had a satisfactory water resistance. One limitation of this process is the relatively high hot-pressing temperature, and, therefore, the focus on the future research can be aimed at reducing it with polymerization catalysts or cross-linkers.

## Figures and Tables

**Table 1:** Depolymerization conditions.

Solvent/Sample	KOH, g L^−1^	BOB: Solvent, g L^−1^	Temperature, °C
MeOH	41.5	100	66
EtOH ^1^	29.2	100	80
i-PrOH	41.5	100	80
BuOH	41.5	100	80

^1^ ethanol-water solution 1:10 (m/m).

**Table 2:** Variable factor levels for DOE.

Variable	Factor Level	
	Low	High
c, wt%	20	40
T, °C	200	250
t, min	2	8

**Table 3:** Duration of the hot-pressing cycles.

Cycle	t = 2 min	t = 8 min
1	3.5 MPa (1 min 20 s)	3.5 MPa (2 min 30 s)
	0.1 MPa (10 s)	0.1 MPa (30 s)
2	1.7 MPa (20 s)	1.7 MPa (2 min)
	0.7 MPa (10 s)	0.1 MPa (30 s)
3	-	1.7 MPa (2 min)
	-	0.7 MPa (30 s)

**Table 4:** Chemical properties of SA-based adhesive samples.

Sample	Acid Number, mg KOH g^−1^	Epoxy Groups, mmol g^−1^	Soluble Substance in THF, wt%	Ash Content, wt%
MeOH	70.9	0.45	44.0	12.9
EtOH	95.8	0.61	51.8	6.7
i-PrOH	122.0	0.11	58.1	9.3
BuOH	91.1	0.25	57.5	6.6

**Table 5:** Molecular weights of adhesive samples.

Sample	M_n_, kDa	M_w_, kDa	M_w_/M_n_
MeOH	9.886	7526	761
EtOH	9.036	6577	728
i-PrOH	3.926	1600	407
BuOH	10.502	7525	717

**Table 6:** Estimations of chemical composition (dry-basis) of adhesive samples from TGA analysis.

Sample	Cellulose, wt%	Aromatic Suberin + Lignin, wt%	ω-Hydroxy Acids, wt%	α, ω-Diacids, wt%
MeOH	9.2	20.3	12.2	13.3
EtOH	9.0	21.4	17.5	11.9
i-PrOH	11.3	22.3	13.8	13.9
BuOH	8.3	22.1	14.2	11.2

**Table 7:** Mechanical properties of particle boards obtained from MeOH, EtOH, i-PrOH and BuOH adhesives.

Adhesive_c	MOE, N mm^−2^	MOR, N mm^−2^	TS 24 h, %	Density, g cm^−3^
MeOH_20	1690	6.18	32.1	0.847
MeOH_30	1211	5.65	24.5	0.857
MeOH_40	1572	5.19	19.0	0.884
EtOH_20	2266	8.39	23.6	0.855
EtOH_30	2331	7.79	14.7	0.867
EtOH_40	2040	6.99	10.1	0.864
i-PrOH_20	2866	9.55	16.1	0.860
i-PrOH_30	2862	10.17	10.9	0.869
i-PrOH_40	2459	8.23	8.0	0.864
BuOH_20	2036	5.96	14.3	0.880
BuOH_30	1934	6.26	11.7	0.868
BuOH_40	1292	4.98	7.4	0.869
EN 312 P3 ^1^	≥2050	≥15	≤17	–
EN 312 P2 ^2^	≥1600	≥11	–	–

^1^ Standard requirements for non-load-bearing boards (>6–13 mm thick) for use in humid conditions. ^2^ Standard requirements for boards (>6–13 mm thick) for interior fitments (including furniture) for use in dry conditions.

**Table 8:** Properties of particle boards, depending on the hot-pressing parameters.

Variable Parameters			MOE, N mm^−2^	MOR, N mm^−2^	TS 24 h, %	Density, g cm^−3^
c, wt%	T, °C	t, min				
20	210	2	659	2.08	41.8	0.688
20	210	8	1831	6.63	22.5	0.854
20	250	2	2820	7.64	17.3	0.845
20	250	8	3518	11.44	3.9	0.846
40	210	2	1403	5.45	20.9	0.844
40	210	8	2187	7.77	12.4	0.891
40	250	2	2612	7.92	8.3	0.880
40	250	8	2723	10.31	2.1	0.853

**Table 9:** Characteristics of particle boards, depending on the pressing parameters (additional points).

Variable Parameters			MOE, N mm^−2^	MOR, N mm^−2^	TS 24 h, %	Density, g cm^−3^
c, wt%	T, °C	t, min				
29	232	5.63	2709	9.51	11.9	0.894
40	219	5.00	2149	7.07	12.3	0.876
29	224	5.90	2453	8.35	14.0	0.883
29	217	4.70	2073	7.34	18.6	0.863
33.5	230	2.00	1593	5.49	19.2	0.850
39	240	5.03	2679	8.74	6.9	0.896
20	233	5.30	2840	9.03	15.6	0.889
30	250	3.59	2951	8.76	7.0	0.870

**Table 10:** Constraints for hot-pressing parameters and response values.

Value	Goal	Lower Limit	Upper Limit
c, wt%	is in range	20	40
T, °C	minimize	210	250
t, min	minimize	2	8
MOE N, mm^−2^	maximize	1800	3917
MOR N, mm^−2^	maximize	11.00	12.59
TS 24 h, %	minimize	1.88	44.94
Density, g cm^−3^	is target = 0.83	0.671	0.952

**Table 11:** Mechanical properties of particle boards obtained under optimal conditions.

Response Value	Result	95% Prediction Interval Lower Limit	95% Prediction Interval Upper Limit
MOE, N mm^−2^	3833	3249	3939
MOR, N mm^−2^	11.27	10.17	12.55
TS 24 h, %	6.26	4.49	7.12
Density, g cm^−3^	0.903	0.837	0.913
IB, N mm^−2^	1.33 ^1^	-	-

^1^ EN 312 P2 requirement ≥ 0.40 N mm^−2^.

## Data Availability

Not applicable.
